# Forcing of anthropogenic aerosols on temperature trends of the sub-thermocline southern Indian Ocean

**DOI:** 10.1038/srep02245

**Published:** 2013-07-22

**Authors:** Tim Cowan, Wenju Cai, Ariaan Purich, Leon Rotstayn, Matthew H. England

**Affiliations:** 1 CSIRO Marine and Atmospheric Research, Aspendale, Victoria, Australia; 2 Australian Research Council Centre of Excellence in Climate System Science, University of New South Wales, New South Wales, Australia

## Abstract

In the late twentieth century, the sub-thermocline waters of the southern tropical and subtropical Indian Ocean experienced a sharp cooling. This cooling has been previously attributed to an anthropogenic aerosol-induced strengthening of the global ocean conveyor, which transfers heat from the subtropical gyre latitudes toward the North Atlantic. From the mid-1990s the sub-thermocline southern Indian Ocean experienced a rapid temperature trend reversal. Here we show, using climate models from phase 5 of the Coupled Model Intercomparison Project, that the late twentieth century sub-thermocline cooling of the southern Indian Ocean was primarily driven by increasing anthropogenic aerosols and greenhouse gases. The models simulate a slow-down in the sub-thermocline cooling followed by a rapid warming towards the mid twenty-first century. The simulated evolution of the Indian Ocean temperature trend is linked with the peak in aerosols and their subsequent decline in the twenty-first century, reinforcing the hypothesis that aerosols influence ocean circulation trends.

Ocean warming is a key signature of climate change, with the oceans accounting for more than 90% of the Earth's warming since the 1950s^1^. Warming in the upper 700 m over 1960–1999 ranges from 0.04–0.06°C per decade for the North Atlantic to less than 0.02°C per decade for the Indian and Pacific Oceans^2^. About 30% of the recent warming^3^ has occurred below 700 m, while around 65% of upper-ocean warming has occurred in the tropical Atlantic and Pacific^4^. Away from the polar latitudes the pattern of change is one of warming across the Southern Hemisphere (SH) surface waters, extending down to 600 m in the extratropics (~40°S–50°S) (Fig. 1). The opposite has occurred in the thermocline and sub-thermocline waters of the SH tropics and subtropics which have cooled over the late twentieth century^5^,^6^,^7^ (Fig. 1). The strongest cooling trend is seen in the tropical Indian Ocean (IO) (Fig. 1b), extending into the subtropics to a depth of around 900 m, which has coincided with a decrease in sea-level in the southern tropical IO^8^. The subsurface cooling in the Indo-Pacific dominates the global ocean pattern, and involves peak amplitude in both thermocline and sub-thermocline waters (Fig. 1d).

The dynamics of the IO thermocline cooling is still a topic of debate. Proposed processes include decadal variability^9^, a shoaling of the western Pacific thermocline induced by a weakening of the easterly trade winds^5^, a southward shift and spin-up of the subtropical gyre^5^,^6^,^9^,^10^, as well as a direct influence from the Indonesian Throughflow (ITF), wherein the Pacific transmits a coastally trapped oceanic upwelling Rossby wave to the tropical IO^5^,^11^,^12^. The subtropical gyre shift and spin-up, which is linked to enhanced Ekman pumping velocity and a shoaling thermocline^7^,^9^,^13^, is associated with enhanced regional IO Walker and Hadley cells^8^. The subtropical gyre shift coincides with a poleward shift and strengthening of the midlatitude westerlies, and is attributed to a combination of stratospheric ozone depletion and increased greenhouse gases (GHGs)^14^. Another potential factor is the weakening of the Pacific Walker circulation, which may be forced by both GHG emissions and natural variability^15^,^16^. However, while some modelling studies suggest the Pacific had very little impact on the subsurface IO prior to the 1990s^9^,^17^, future surface warming in the tropical and subtropical Pacific may lead to wind-induced redistributions of upper ocean water and a shoaling thermocline in the southern IO^18^.

Anthropogenic forcings, such as GHGs have already been attributed as the major cause of historic ocean warming^19^. Aside from GHGs, anthropogenic aerosols (AAs) have been implicated in forcing changes in the large-scale climate, such as the south Asian and East Asian monsoon^20^,^21^,^22^ and North Atlantic sea surface temperatures (SSTs)^23^, including cross-hemispheric changes in the tropical atmospheric circulation^24^,^25^,^26^. In addition, natural aerosols (e.g., sea salt, dust) are also thought to have forced changes to the surface radiation balance and temperatures in the Arabian Sea^27^, leading to a late twentieth century increase in pre-monsoonal tropical cyclones^28^. For the sub-thermocline tropical and subtropical IO, it has been postulated that AAs such as sulfates could have forced the cooling^6^,^29^,^30^. Specifically, model simulations of an AA-induced response show a strengthening in the Atlantic Meridional Overturning Circulation and an increase in the cross-hemispheric transport of heat from the SH to the Northern Hemisphere (NH) via the global conveyor^29^. As a result, heat is removed from the subtropical latitudes of the southern IO sub-thermocline, and is transported to the North Atlantic, increasing the stability of North Atlantic Deep Water formation^29^. Model simulations indicate that this process is likely to reverse in the mid-twenty-first century as AAs decrease in the atmosphere and GHGs continue to rise^30^,^31^.

Toward the end of the twentieth century the subsurface tropical IO exhibited a rapid warming^12^, which coincided with a shift to the negative phase of the Pacific Decadal Oscillation (PDO). This rapid warming also coincided with a reversal of the weakened equatorial Pacific easterly winds and a strengthening of the ITF^9^,^32^, as well as a sea level increase over the western tropical Pacific^33^. In the context of understanding the causes of the sub-thermocline temperature changes, we use a suite of Coupled Model Intercomparison Project phase 5 (CMIP5) models forced with all historical radiative forcings and as well as individual forcing runs. We use these to (i) examine whether the sub-thermocline cooling and/or rapid temperature trend reversal (warming) of the tropical-subtropical IO is anthropogenic or naturally forced; and (ii) assess future projections of the sub-thermocline temperatures to the mid twenty-first century from available model output.

## Results

### Observed late twentieth century trends in IO sub-thermocline temperature

**Figure 1:**
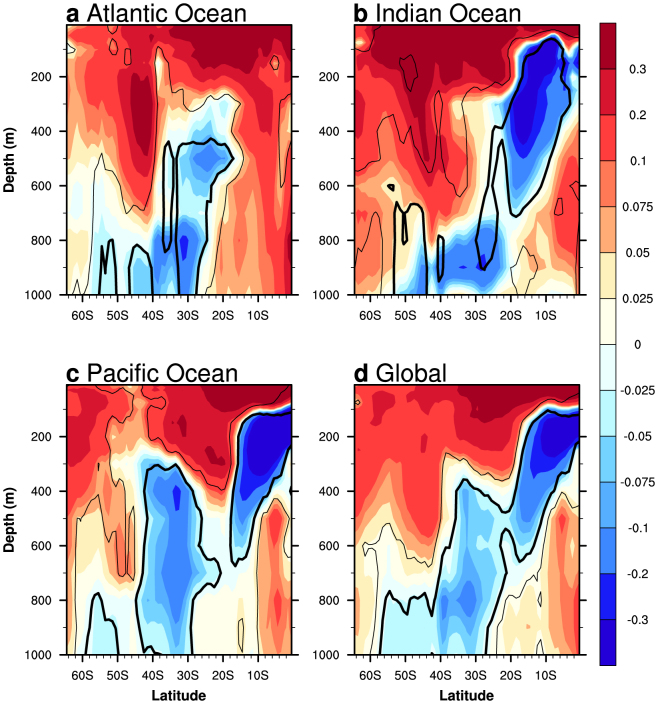
Observed zonal-mean linear subsurface temperature trends over 1960–1999 in the SH ocean basins. Zonally-averaged linear trends over 1960–1999 of subsurface temperature over the southern tropical and subtropical: (a) Atlantic Ocean (70°W–20°E), (b) Indian Ocean (40°E–110°E), (c) Pacific Ocean (120°E–70°W), and (d) global ocean. Trend units are °C 40-years^−1^. The observational trend estimates are based on an average of three products: Levitus09^55^, Ishii09^56^ and SODA-POP V2.2.4^57^, while the average for the Indian Ocean also includes an estimate from the Indian Ocean Thermal Archive (IOTA)^34^. Levitus09 observations are confined to the upper 700 m only, below that the estimate is based on Ishii09 and SODA-POP only. Significant trends at the 95% confidence level, based on a *t*-test for Levitus09, Ishii09 and SODA-POP, are shown within the contours. While IOTA is included in the trend average in (b), the original source data was unavailable for significance testing (only trend data were provided), therefore IOTA is excluded in the significance test.

The cooling of the tropical and subtropical subsurface IO over the late twentieth century (1960–1999) is evident in a number of observational products, including the Indian Ocean Thermal Archive (IOTA)^34^ (Fig. S1). All gridded observational products show good agreement with cooling trends in excess of 0.3°C 40-years^−1^, which extends off the equator (~10°S) at 100 m, to below 800 m in subtropical waters (~30°S–40°S). The tropical region of the southern IO cooling pattern is slightly broader and stronger than in the other ocean basins (Fig. 1). The entire southern IO (averaged over 40°E –110°E) exhibits a substantial surface warming, and a strong deep warming in the extratropics (40°S–50°S) that extends down to around 600 m^5^,^10^,^35^. A similar warming is also seen in the southern Atlantic and Pacific Oceans (Figs. 1a, c). The upper 1000 m temperature trends from the tropical-extratropical IO are consistent with observed and assimilated oceanic heat content trends for the top 2000 m for the second half of the twentieth century^1^,^3^. The one inconsistency is the lack of an extratropical warming in SODA-POP, which may be due to the scarcity of observations in the regions south of 35°S^13^.

### Simulated trends in CMIP5 climate models

**Figure 2:**
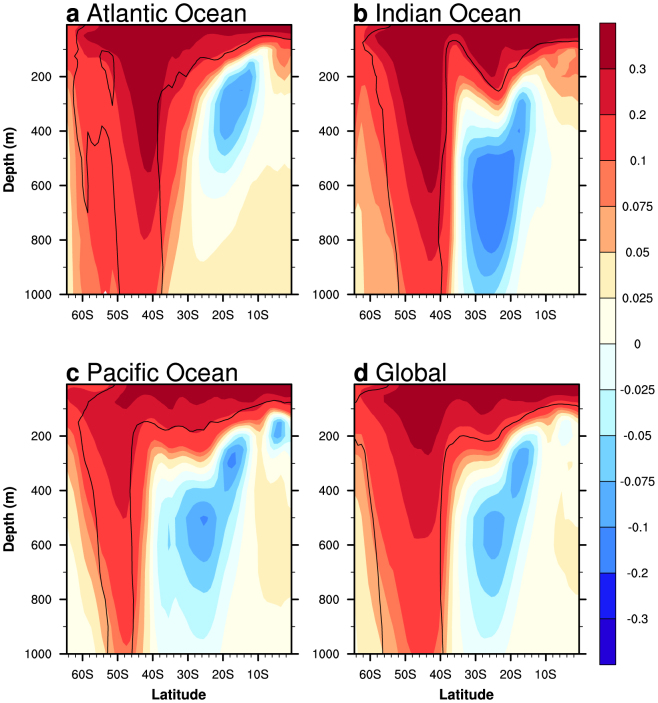
Simulated zonal-mean linear subsurface temperature trends in the SH ocean basins. Simulated zonally-averaged linear trends from a 42 CMIP5 model ensemble (one experiment each) over 1960–1999 of subsurface temperature over the southern tropical and subtropical: (a) Atlantic Ocean (70°W–20°E), (b) Indian Ocean (40°E–110°E), (c) Pacific Ocean (120°E–70°W), and (d) global ocean. Trend units are °C 40-years^−1^. Significant trends, greater than one-standard deviation of the inter-model spread, are shown within the contours. Although the cooling trends are not significant based on the model spread, they are statistically significant at the 95% confidence level, based on a *t*-test on the 42 simulation ensemble (not shown in plot).

Previously it was reported that CMIP3 models show good agreement between the trend pattern of the subsurface IO temperatures and the IOTA observational estimate^5^,^6^. Applying the same technique to 42 CMIP5 models (Table S1; taking one historical experiment per model for the 1960–1999 period), the simulated multi-model ensemble (MME) trend pattern for each ocean basin is calculated (Fig. 2). All basins exhibit a sub-thermocline cooling (warming) in the tropical (extratropical) waters, with the IO exhibiting the greatest magnitude of change (Fig. 2b). The extratropical warming in all ocean basins is deeper than the observational and assimilated estimates (*c.f.* Fig. 2 and Fig. 1), despite the observed average IO warming (Fig. 1b) taking into account the IOTA estimate, which shows the deepest penetration (~800 m) of warming in all four estimates (Fig. S1). The larger, deeper warming may result from an overly weak stratification, allowing more heat to be transferred to the deeper ocean^10^, or stronger poleward shifts in the subtropical IO gyre, as seen in CMIP3 models^5^. Overall, the CMIP5 models show a fairly good comparison in their extratropical IO warming (Fig. 2b).

A southward shift of the subtropical IO (thermal) gyre of between 0.5–0.75° accounts for much of the temperature changes observed in the southern subsurface IO^10^. This is yet to be assessed in CMIP5 models. The degree to which unforced model drift can account for the trends is examined in a 15 model MME (see Methods). While the overall magnitude of the subtropical warming is slightly reduced in the de-drifted MME, the locations of the warming and cooling centres are not substantially influenced by the model drift (Fig. S2). The de-drifted MME shows a stronger sub-thermocline cooling in all basins, including the IO (Fig. S2b), where models show good agreement (Fig. S3). The de-drifted result confirms that in the upper ocean (above ~1500 m) forced trends dominate over model drift, as shown in CMIP3 models^36^.

### Rapid warming of the sub-thermocline Indian Ocean

**Figure 3:**
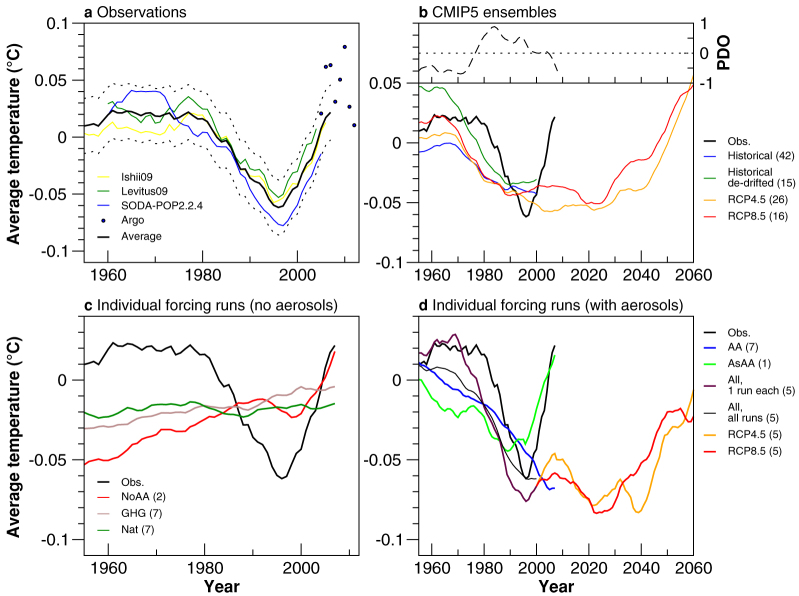
Average sub-thermocline temperature in the southern Indian Ocean. Mean sub-thermocline temperature across the southern tropical and subtropical IO from: (a) observations, (b) CMIP5 historical and RCP experiments ensembles, (c) individual forcing experiment ensembles from runs that contain external forcings other than anthropogenic aerosols, and (d) individual forcing experiment ensembles from runs that contain anthropogenic aerosols, including the RCP4.5 and 8.5 experiments. The number of models used in each MME is shown in the brackets of the figure legend. The models selected to show the RCP changes in (d) are the CMIP5 models that have individual forcing experiments. The region averaged for the observations is 40°E–110°E, 10°S–30°S, 300–900 m (except for Levitus09 which is 300–700 m), and for simulations is 40°E–110°E, 15°S–35°S, 300–900 m. As the models simulate the cooling further south, the latitudinal extent of the simulations is extended 5° polewards. A time series of the PDO^59^, with a 144-month low-pass filter, is shown in (b). The observational estimate average is based on four observed products: Levitus09 (1955–2009), Ishii09 (1945–2011), Argo^58^ (2005–2012) and SODA-POP V2.2.4 (1955–2010). All time series have been low-pass filtered using an 11-year running mean, except for the Argo measurements which show the interannual variability. The dotted lines in (a) indicates the error estimate of the observational average based on the one-standard deviation of the Argo measurements (considered true observations).

Observational estimates show the cooling trend in the tropical sub-thermocline IO reversed in the mid-1990s (Fig. 3a), returning to pre-1980 temperatures in around a decade. This also coincided with an accelerated warming in the south Atlantic sub-thermocline depths (Fig. S4a). Argo measurements reveal the recent interannual variability, confirming that the warming is not spurious (Fig. 3a, dots). Due to the lack of observations prior to the 1950s, it is unclear whether a trend reversal of such magnitude has occurred in earlier decades; however recent evidence suggests that this trend reversal may be a result of a wind regime shift in the Pacific^12^. The switch to a positive PDO phase in the late 1970s aligns with the sharp decline in IO temperatures during this period (Fig. 3b), however the lack of a warming prior to the 1980s (negative PDO phase) suggests that external forcings may have also played a role. In the Pacific, a rapid warming occurs in the late 1980s and then temperatures dip again before the end of the twentieth century, evidence of strong multi-decadal variability (Fig. S4c).

The extent to which anthropogenic climate change contributes to the subtropical IO temperature trend reversal is examined using a 42 CMIP5 MME, averaged over 300–900 m (Fig. 3b). The results clearly show a cooling from around 1970, which slows down in the beginning of the 1990s (Fig. 3b, blue curve). This is seen more clearly in the de-drifted MME (15-models) curve (Fig. 3b, green curve) with temperatures flattening in the post-1990 decade. Both the magnitude of the observed cooling and trend in the de-drifted MME (historical) is more than 10 times larger than the standard deviation of the 15-model pre-industrial control run mean (Fig. S5). Thus, the observed cooling is extremely unlikely to be due to naturally-forced decadal variability alone, and is more likely externally forced.

Future projections of the evolving state of the subtropical sub-thermocline IO temperature, using two Representative Concentration Pathway (RCP) MMEs (RCP4.5 (medium-low forcing), 26 models; RCP8.5 (high forcing), 16 models), produces a period of relative temperature stability during the early part of the twenty-first century (Fig. 3b). In the RCP4.5 MME, the cooling ceases in the mid 2000s and is proceeded by a 30-year temperature hiatus, before a warming commences around 2030, which increases rapidly to about 0.05°C per decade between 2040–2060 (Fig. 3b, orange curve). In the RCP8.5 MME the period of temperature hiatus is similar in duration, with the rapid temperature rise commencing earlier, around the mid-2020s (Fig. 3b, red curve). The temperature hiatus in the other ocean basins is shorter in duration; the Pacific and Atlantic show rapid warming at the start of the twenty-first century (Fig. S6). Why the IO exhibits a delayed warming is one focus of this study.

To summarise thus far, the results highlight three main findings for the southern IO:

- the pre-1990s IO sub-thermocline cooling is most likely due to climate change and not natural variability;
- the models show a slowdown in the cooling trend during the 1990s; and
- simulate a rapid warming after 2020, that lags observational estimates by approximately 30 years.

### The role of external forcings in the IO cooling and warming

To understand the role of individual forcings in driving sub-thermocline trends we analyse output from five CMIP5 models and two CMIP3 models for which such targeted experiments are available (see Table S2 and Methods section). Using a low-resolution coupled model, it has previously been shown that AAs could play a role in the observed cooling trend^6^,^29^; as such model experiments that do, and do not, contain an anthropogenic aerosol forcing are placed into subgroups. First, a comparison between the no anthropogenic aerosol (NoAA), well-mixed greenhouse gases only (GHG) and natural forcings only (Nat) MMEs is undertaken (Fig. 3c). The NoAA and Nat MMEs exhibit a small dip in the IO sub-thermocline temperature: for the Nat MME, this occurs between the late 1970s and the 1980s, while for the NoAA MME, this occurs during the 1990s. The slight cooling in the Nat ensemble is predominantly contributed to by the CMIP3 models (Figs. S7f and g), and may reflect the impact of stratospheric aerosols (possibly due to the El Chichón volcanic eruption in 1982) on oceanic heat content and sea level rise^2^,^37^. The short 10-year cooling period in the NoAA MME, however, occurs around 1990 and mainly reflects the cooling in the IPSL-CM5A-LR ensemble (Fig. S7c). As the cooling is not replicated in the two NoAA models' corresponding Nat ensembles, the cooling seems unlikely to be a response to the 1991 Mt Pinatubo volcanic eruption^37^. Despite lacking a long-term cooling trend, the NoAA MME exhibits an accelerated warming in the subtropical sub-thermocline in the late 1990s (Fig. 3c, red curve), consistent with observational estimates. The NoAA MME includes a stratospheric ozone depletion forcing, which complements the role of GHGs in that it also induces a poleward shift in the SH oceanic supergyres^14^. The GHG MME, on the other hand, shows a near linear warming throughout the entire late twentieth century (Fig. 3c, light brown curve). This initially suggests that GHGs alone are unlikely to force the sub-thermocline cooling; however in analysing the MME trend pattern (five CMIP5 + two CMIP3 models) we see an isolated cooling between 20°–30°S in the southern IO, from 500–1000 m (Fig. S8). This cooling is not seen in the other ocean basins, and adds weight to the hypothesis that a GHG-induced poleward shift of the subtropical IO supergyre is associated with a sub-thermocline cooling^5^,^10^. However, separating the individual CMIP5 and CMIP3 models indicates a lack of consensus in a GHG-induced sub-thermocline trends (Fig. S7, orange curves). Three out of five CMIP5 models simulate a GHG-induced sub-thermocline cooling trend from 1950–2005, whereas the two CMIP3 show mixed results (i.e., the GFDL-CM2.1 exhibits strong multi-decadal variability).

The IO sub-thermocline cooling is best represented by MMEs incorporating human-generated aerosols (Fig. 3d) – including an anthropogenic aerosol only (AA) MME and a 5-member ensemble from the CSIRO-Mk3.6 that contain all forcings, except that anthropogenic aerosol emissions are fixed at 1850 levels outside Asia (AsAA). The AA ensemble generates a strong cooling that only slows slightly after 2000 (Fig. 3d, blue curve), with strong inter-model consensus (Fig. S7, blue curves). Focusing on the AA-induced MME trend patterns across all SH ocean basins we see that AAs induce a substantial cooling that penetrates down below 1000 m (Fig. S9). Despite this, the subtropical sub-thermocline IO shows a weak warming (Fig. S9b), however on closer inspection of individual model trends, the warming predominantly comes from one model: CanESM2. This model also tends to generate an aerosol-induced cooling of the North Atlantic Deep Water Formation region (associated with a weakening of the Atlantic Meridional Overturning Circulation), opposite to what is simulated in other models forced by AAs^30^. At least three models (CanESM2, IPSL-CM5A-LR, GFDL-CM2.1) show a temperature trend reversal in their AA ensembles, towards the end of the twentieth century. The AA-induced cooling is seen across all of the SH ocean basins, however it is much more variable in the IO (Fig. S10). The AsAA curve shows a substantial cooling from 1960–1990, and then follows the warming trajectory of the no-aerosol runs (NoAA, GHG and Nat, Fig. 3d, light green curve). The warming after 1990 in AsAA is unlikely to be due to the decline of Asian aerosols, as sulfur dioxide (the main precursor to sulfate aerosols) emissions from East Asia continued to rise post-2000^38^. The lack of cooling in AsAA beyond 1990 could also reflect the lack of non-Asian aerosols (held at pre-industrial levels) that would enhance any cooling trend and/or the emergence of other forcings such as GHGs that may induce a top down warming of the upper ocean. Remarkably, the five-member all forcings (All) ensemble that includes only the five CMIP5 models used in this individual forcings assessment (one run each; see Table S2) simulates the cooling and timing of the rapid warming with great accuracy (Fig. 3d, maroon curve), although the rate of warming during 1995–2012 is less than observed. Replacing the one-run All ensemble with all ensemble members from the five CMIP5 individual forcing models again reproduces the late century cooling with similar accuracy (Fig. 3d, thin black curve). Extending these historical simulations to 2060 with the RCP4.5 and 8.5 projections produces a similar evolution (hiatus period with no long-term cooling or warming) until 2030–2040, followed by a rapid warming (Fig. 3d, orange and red curves).

### The role of surface winds and natural variability

The observed long-term cooling trend in the southern sub-thermocline IO can only be explained in the MMEs which include both anthropogenic and natural forcings (i.e., it is unlikely to be a result of internal model variability; Fig. S5). Previous assessments have hypothesised on the role of the wind in driving tropical IO upper thermocline changes^8^,^18^. We therefore analyse the wind stress and resultant curl changes induced by GHGs and AAs (from the five CMIP5 models, excluding the two CMIP3 models due to lack of data). The results show that AAs induce only a slight weakening of the midlatitude westerlies, which contributes to a weak negative curl at 40°S (Fig. S11b); this has very little bearing on the sub-thermocline changes through the Ekman suction, which can force a shoaling thermocline^18^ (figure not shown). On the other hand, GHGs induce a stronger wind stress change (stronger westerlies and southeast trades), which induces both a strong positive (negative) curl trend in the extra-tropical (subtropical) IO (Fig. S11a). The GHG-induced change also corresponds with a large-poleward shift and spin-up in the super gyre, which pumps heat into the IO midlatitudes and takes heat out of the subtropical sub-thermocline, as a result of the Ekman pumping velocity^5^,^18^. This implies that the wind-driven circulation plays a crucial role in southern IO changes under the forcing of GHGs. However, AAs, due to their hemispheric imbalance, force change through oceanic readjustment^29^.

The rapid warming at the turn of the twenty-first century is more difficult to attribute, due to the number of possible contributing factors. These include natural fluctuations such as the PDO, a lack of large tropical volcanic eruptions, a peak and decline in global AAs, and increasing GHGs. Models show difficulty in simulating the observed warming after the mid-1990s, which points toward the greater role that the PDO possibly plays in the IO temperature reversal^12^, particularly after the 1980s^9^,^17^. The mismatch between the models and observations may be associated with either the performance of each model (i.e., getting the right sign of change for the correct reasons) or each model's internal variability, which will not necessarily match the variability phase in the observational record. The rapid warming in the Atlantic occurred around the same time (Fig. S4a), suggesting the possibility of a similar forcing between ocean basins, although the MME performs better at simulating the timing of warming in both the Atlantic and Pacific Oceans.

## Discussion

While future emissions of AAs remain uncertain^39^,^40^, recent studies suggest sulfur dioxide peaked in the late twentieth century across many regions of the NH, such as the United States and Europe^38^,^41^. In China, sulfur dioxide emissions rose until 2006, and have declined since then, impacting global totals^42^,^43^. Emissions used in CMIP5 show sharp decreases in sulfur dioxide and carbonaceous aerosols from the start of the RCPs, i.e., after 2005^44^. For sulfur, the observed decline after 2005 is generally consistent with the forcing applied in the RCPs^44^.

A sub-sample of 12 CMIP5 models that have available temperature and aerosol information (see Table S1) simulate the peak in aerosol loading between the late twentieth and early twenty-first centuries. The evolution of all aerosols (anthropogenic and natural) can be measured by the mean aerosol optical depth (AOD) at 550 nm averaged across the NH (Fig. S12). We take the average across the NH as this is the hemisphere where most aerosols are emitted from anthropogenic sources^45^. Each model simulates different AOD peak values and subsequent declines throughout the twenty-first century (Fig. S12, black lines). For example, the CSIRO-Mk3.6 model (based on a 10-run ensemble), shows a peak in the late-1980s of around 0.26 followed by a sharp decrease in the AOD of −0.084 (dimensionless) from 2006–2100 (Fig. S12c). Both ACCESS models show smaller peak AOD values, and a much slower rate of decrease (Fig. S12a,b). The differences in the AOD between the CMIP5 models reflect the complexity and uncertainty of aerosol processes^45^. Note that the AODs (in Fig. S12) include a substantial contribution of natural aerosols, such as dust and sea salt – these can also affect the magnitude and timing of the AOD peak.

**Figure 4:**
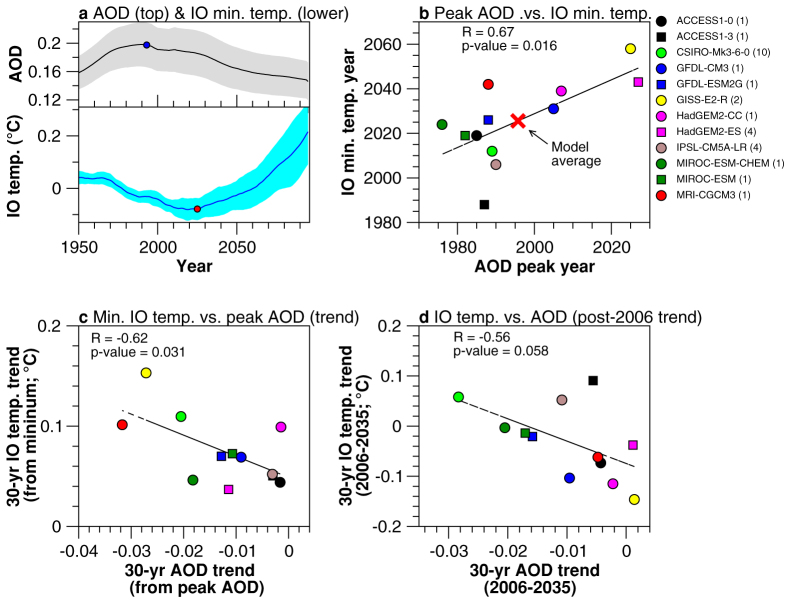
Southern Indian Ocean sub-thermocline warming versus aerosol optical depth. (a) Average temperature of the southern sub-thermocline IO (bottom plot; blue line) versus aerosol optical depth (AOD) at 550 nm averaged over the NH (top plot; black line) from 1950–2100 (based on a 12 CMIP5 MME, including historical and RCP8.5 experiments; see Table S1), (b) scatterplot of the year at which the southern sub-thermocline IO temperature reaches a minimum versus peak NH AOD, for the 12 CMIP5 models, (c) scatterplot of 30-year trend in southern sub-thermocline IO temperature after minimum versus 30-year trend in NH AOD after peak value, and (d) scatterplot of 30-year trend in southern sub-thermocline IO temperature from 2006–2035 (i.e., beginning of RCP8.5 experiment) versus 30-year trend in NH AOD from 2006–2035. The number of runs that make up each point are listed in the brackets after model name. The years shown in (a) and (b) and trends in (c) and (d) are based on 11-year running means (to remove year-to-year noise). The line-of-best-fit, R and p-values are also shown. The inter-model spread in (a) is based on the 95% confidence interval. The regression lines in (b) and (c) are statistically significant at the 95% confidence level, whereas the regression line in (d) is significant at the 90% confidence level.

To elucidate an influence of aerosols on the IO warming, for each model we compare the year of the mean sub-thermocline IO temperature minimum (see Fig. S12, red dots) against the peak AOD year (Fig. S12, blue dots). A MME of 12 CMIP5 models shows the AOD and IO temperature evolution at the turn of the century, with aerosols peaking prior to the year 2000, while the IO reaches a temperature minimum after 2020 (Fig. 4a). Individual models with an early AOD peak simulate an early reversal of temperature (except ACCESS1-3), with seven out of 12 models simulating an IO temperature minimum before 2030 (Fig. 4b). Four models (GISS-E2-R, HadGEM2-ES, HadGEM2-CC, GFDL-CM3) simulate an AOD peak after the year 2000 and show a temperature minimum after 2030. A possible reason for GISS-E2-R showing a delayed peak in AOD is that it includes nitrate aerosols from agricultural emissions of ammonia, which are projected to increase in the RCPs^44^. However, historical nitrate increases in GISS-E2-R are also overly strong^46^, so the effect may be over-estimated. HadGEM2-ES has a broad maximum in global-mean AOD, with little change between roughly 1990 and 2030 (based on 11-year smoothing). This may reflect changes in natural aerosols (dust and sea salt). In terms of the model-average mean IO temperature, the minimum occurs around 2025, just over 30-years after peak AOD (in 1995) (Fig. 4b, red cross). We next test whether the magnitude of the AOD decline after peak value is a factor in determining how quickly the IO warms after the minimum temperature is reached (i.e., the end of the sub-thermocline cooling). The results highlight the strong, statistically significant relationship between the magnitude of the AOD decline and the IO sub-thermocline warming (Fig. 4c). Models that show a smaller AOD decline over the 30-year period post AOD peak tend to simulate a smaller future warming in the IO. One model however shows no sustained warming in the twenty-first century (IPSL-CM5A-LR, Fig. S12), so caution should be noted for this model. Looking at the post-2006 period, one can see that a similar relationship exists between the magnitude of the AOD change and the IO sub-thermocline temperature. Models with little or no change to the AOD post-2006 (i.e., the models that have broader AOD peaks) tend to show continued cooling of the IO temperature after 2006 (Fig. 4d). Models with a sharper peak in their AOD evolution show weaker changes in the IO temperatures after 2006 (Fig. S12).

The above results highlight a number of key points. Firstly, the models tend to show a longer than observed lag between peak aerosol (based on the AOD) and the end of the IO cooling of around 30 years.The observed rapid warming occurred in the mid-1990s, while global sulfur emissions (the main constituent of sulfates) peaked in the late 1970s^38^. This is substantially due to a reduction in emissions from non-Asian regions^41^,^45^, and coincided with a European and United States warming from the 1980s^47^,^48^. Based on the assumption that the observed IO warming is closely associated with the decline in sulfate aerosols, the observed lag is between 15–20 years, about 10–15 years less than the lag in the models. Secondly, the magnitude of the warming after the temperature minimum is proportional to the magnitude of the AOD reduction, suggesting the pace at which the models remove the aerosols may dictate how quickly the IO warms. Finally, models with no well-defined AOD peak tend to show a more protracted IO cooling post-2006, which again reinforces that the rate of aerosol decline is an important factor.

The role of GHGs adds to the uncertainty in this analysis. A subsurface cooling can be generated through a poleward shift in the SH circulation as a result of increasing GHGs^5^,^10^. Modelling results from the CSIRO-Mk3.6 also suggest that GHGs weaken the ITF^30^, while the Atlantic Meridional Overturning also declines in strength under enhanced CO_2_ forcing^31^, implying less cooling in the southern subtropical latitudes. GHGs have also been implicated in forcing surface wind changes across the tropical IO; however this is not seen in a five CMIP5 MME (Fig. S11a). Using observational analyses, studies have speculated that changes in the tropical winds are associated with “invigorated” local Hadley and Walker cells^8^,^49^, leading to a drop in sea levels in the tropical southern IO^50^. However, models forced with GHGs induce a weakening of the (Pacific) Walker circulation^51^ and a weakening and expansion of the (globally defined) Hadley circulation^52^, highlighting the uncertainty in using different observational analyses datasets^25^. While individual model GHG-only ensembles show no strong consensus for the broader sub-thermocline IO (Fig. S7), a weak localised sub-thermocline cooling between 20°S–30°S (in the MME) (Fig. S8) can be attributed to GHGs, generated by strong wind-stress curl changes in the IO midlatitudes (Fig. S11a).

In summary, the observed cooling of the sub-thermocline tropical-subtropical IO is most likely due to a combination of anthropogenic (both AAs and GHGs) and natural forcings (volcanic eruptions), and unlikely due to variability in the PDO alone (assuming the PDO is not a manifestation of climate change). The two models that exclude AAs from their simulations exhibit a long-term warming in this region, consistent with the notion that AAs induce a cooling through an intensification of the global conveyor, which results in heat being removed from the IO and delivered to the North Atlantic^6^,^29^. This process is yet to be tested using the CMIP5 models. The observed rapid warming of the IO sub-thermocline waters in the mid-1990s coincides with a phase shift of the PDO^12^, as well as sea-level variations and wind changes in the Pacific^32^,^33^,^50^,^53^. A CMIP5 MME simulates a reduction in the rate of cooling during this decade, but not a clearly defined warming signal. The discrepancy between the models and the observations may be due to simulated internal variability differences or due to the internal decadal variability in the Pacific. Future projections suggest a period of temperature stability of around 30–40 years from the end of the twentieth century to the mid twenty-first century, followed by rapid warming. The timing of the commencement of this warming appears dependent on the total change in AA levels; with models exhibiting a strong (weak) decline in future aerosols simulating a greater (weaker) magnitude of warming after the occurrence of peak aerosols. The role of GHGs in forcing sub-thermocline temperature trends in the IO in the future remains to be determined, as a further poleward shift in the subtropical gyre will potentially offset any warming signal that penetrates into the deep ocean from the surface. Whether or not model biases contribute to the above results, as in CMIP3^54^, also remains to be fully tested. What is clear is that as human generated aerosols continue to decline over the coming century, the subsurface ocean circulation will respond accordingly through an acceleration in warming trends.

## Methods

### Ocean temperature data

The trend cross sections in Fig. 1 are based on three different observational datasets: (i) Levitus09 temperature from the World Ocean Database 2009^1^,^55^, which is based on Argo profiles, expendable bathythermographs (XBT), and mechanical bathythermographs (MBT); (ii) Ishii09 temperature^56^, with bias corrected XBT and MBT data; and (iii) Simple Ocean Data Assimilation (SODA)- Parallel Ocean Program (POP) version 2.2.4^57^, which is an assimilated product that is forced using 20th Century Reanalysis V2 winds. Products (i) – (ii) have 1° × 1° resolution, while SODA-POP has 0.5° × 0.5° resolution. All products, except for Levitus09, contain data for the upper 900 m (maximum depth of this study); Levitus09 contains data for the upper 700 m only. Fig. 1b also contains data from the Indian Ocean Thermal Archive (IOTA)^34^, which is a compiled quality-controlled historical Indian Ocean temperature dataset in the upper 1000 m (IOTA is also used for Fig. S1a). For Fig. 3, Levitus09, Ishii09, and SODA-POP are used, as well as annual values from Argo floats (2005–2012) interpolated onto a 1° × 1° grid^58^. Each observational estimate is also vertically interpolated to the Ishii09 resolution. A PDO index^59^, with a 144-month low pass filter is also used to look at multi-decadal relationships.

### Model simulations

#### CMIP5 historical and RCP runs

Both historical and RCP model experiments from the CMIP5 archive^60^ are analysed. These include a single historical (1950–2005) experiment with all forcings, from 42 models, and a single RCP4.5 and RCP8.5 (2006–2099) run from 26 and 16 models, respectively (Table S1). Each model is re-gridded to a common 1° × 1° (lat × lon) grid, and vertically interpolated to the Ishii09 resolution (as for each observational estimate).

#### Individual forcing runs

Runs with and without particular radiative forcings were taken for five CMIP5 models, and two CMIP3 models. The experiments examined include anthropogenic aerosols only (AA), all forcings except for anthropogenic aerosols (NoAA), well-mixed greenhouse gases only (GHG), natural forcings that include the effects of volcanos and solar irradiance (Nat), and all forcings except that anthropogenic aerosol emissions are fixed at 1850 levels outside Asia (AsAA – only in the CSIRO-Mk3.6^25^; Asia is defined as the region from 10°S–40°N, 65°E–150°E). All time series involving individual model forcings (except for RCP4.5 and RCP8.5) have been weighted according to the number of particular model experiments. The models used are shown in Table S2 (supplementary material). The RCP4.5 and 8.5 experiments from the five CMIP5 models are also analysed as a comparison, with one run from each model used (i.e., no weighting involved). These models are chosen because of model availability at the time of writing. While the detection and attribution method allows for the isolation of possible forcings, an important caveat to note is that due to the low number of available runs per model the uncertainty remains high (i.e., models with less ensemble members would have larger uncertainty levels). While the GHG, Nat and AA ensembles are made up of similar run totals (30, 28, and 26 respectively; see Table S2), the NoAA ensemble consists of only nine runs.

#### Accounting for model drift

At the time of analysis, 15 CMIP5 models were able to be de-drifted, which involves subtracting the pre-industrial control run linear trend from the corresponding historical period. We found that this did not alter the structure of the temperature changes (as in Fig. 2). In CMIP3 models, forced trends dominate model drift in the upper 1500 m, while below this level model drift is important^36^. An assessment of CMIP5 model drift is yet to be undertaken.

#### Statistical analysis

Fig. 1 significance is calculated using a *t*-test based on standard error of the observational estimate average (Levitus09, Ishii09 and SODA-POP). IOTA is included in the trend average in Fig. 1b, but excluded from this significance testing due to the absence of original source data; therefore the significance in Fig. 1b is based on the three observational estimates, not four. For Fig. 2, significance is based on trends which are greater than one standard deviation of the model-spread (42 models). In Fig. 3, 11-year running means are shown to highlight the multi-decadal variability. Individual model time-series are shown in Fig. S7. The significance of the subsurface IO cooling in the de-drifted ensemble (Fig. 3b, green line) is tested against the 15-CMIP5 pre-industrial experiment MME (Fig. S5). In this case, we have taken the last 200-years of each pre-industrial period and averaged over the 15-CMIP5 models. For Fig. 3b, we calculated the 95% confidence interval based on the statistics of 10 different MMEs. For this, we randomly selected one model run from each model and created 10 model ensembles. The historical CMIP5 MME time series of the IO subsurface temperature with error bars is shown in Fig. S13.

## Author Contributions

T.C. conceived the study, and wrote the initial draft, in discussion with W.C. The collation of the data was performed by T.C. and A.P. The design and analysis was conducted by T.C., while W.C., A.P., L.R. and M.E. helped in the interpretation of the results and reviewed the manuscript.

## Supplementary Material

Supplementary Information Supplementary material
