# Supplementary material for: Forcing of anthropogenic aerosols on temperature trends of the sub-thermocline southern Indian Ocean
Source: Sci Rep. 2013 Jul 22;3:2245. doi: 10.1038/srep02245 (PMC3718192; doi:10.1038/srep02245)
Supplement: Supplementary Information — Supplementary material [file srep02245-s1.pdf]

Forcing of anthropogenic aerosols on temperature trends of the sub-thermocline southern Indian Ocean

Tim Cowan*[1,2], Wenju Cai[1], Ariaan Purich[1], Leon Rotstayn[1] and Matthew H. England[2]

[1]CSIRO Marine and Atmospheric Research, Aspendale, Victoria, Australia

[2]Australian Research Council Centre of Excellence in Climate System Science, University of New South Wales, New South Wales, Australia.

*Correspondence and requests for materials should be addressed to T.C. (Tim.Cowan@csiro.au)

Supplementary tables and figures

Table S1| CMIP5 coupled models used in this study.

Institution	Model(s)	Historic De-drifted	RCP4.5 (to 2099)	RCP8.5 (to 2099)
Commonwealth Scientific and Industrial Research Organization (CSIRO) & Bureau of Meteorology (BOM)	ACCESS1-0<br>ACCESS1-3	Y<br>Y	Y<br>Y	Y*<br>Y*
Beijing Climate Center, China Meteorological Administration (bcc)	bcc-csm1-1<br>bcc-csm1-1-m	Y<br>-	Y<br>-	-<br>-
College of Global Change and Earth System Science, Beijing Normal University (BNU)	BNU-ESM	-	-	-
Canadian Centre for Climate Modelling and Analysis (CCCma)	CanESM2	-	Y	Y
National Center for Atmospheric Research (NCAR)	CCSM4	Y	Y	Y
Community Earth System Model Contributors (NSF-DOE-NCAR)	CESM1-BGC<br>CESM1-CAM5<br>CESM1-CAM5-1-FV2<br>CESM1-FASTCHEM<br>CESM1-WACCM	-<br>-<br>-<br>-<br>-	-<br>-<br>-<br>-<br>-	-<br>-<br>-<br>-<br>-
Centro Euro-Mediterraneo per I Cambiamenti Climatici (CMCC)	CMCC-CESM<br>CMCC-CM<br>CMCC-CMS	-<br>-<br>-	-<br>-<br>-	-<br>-<br>-
Centre National de Recherches Météorologiques / Centre Européen de Recherche et Formation Avancée en Calcul Scientifique (CNRM-CERFACS)	CNRM-CM5	Y	Y	Y
CSIRO & Queensland Climate Change Centre of Excellence (QCCCE)	CSIRO-Mk3.6	Y	Y	Y*
Institute of Atmospheric Physics, Chinese Academy of Sciences (g2 & s2) and CESS,Tsinghua University (g2 only)	FGOALS-g2<br>FGOALS-s2	-<br>-	Y<br>Y	-<br>-
National Oceanic and Atmospheric Administration Geophysical Fluid Dynamics Laboratory (NOAA-GFDL)	GFDL-CM2p1<br>GFDL-CM3<br>GFDL-ESM2G<br>GFDL-ESM2M	-<br>Y<br>-<br>-	-<br>Y<br>Y<br>Y	-<br>Y*<br>Y*<br>-
NASA Goddard Institute for Space Studies (GISS)	GISS-E2-H<br>GISS-E2-R	-<br>-	-<br>Y	-<br>Y*
Met Office Hadley Centre	HadCM3<br>HadGEM2-CC<br>HadGEM2-ES	-<br>Y<br>Y	-<br>Y<br>Y	-<br>Y*<br>Y*
National Institute of Meteorological Research/Korea Meteorological Administration	HadGEM2-AO	-	-	-

Institution	Model(s)	Historic De-drifted	RCP4.5 (to 2099)	RCP8.5 (to 2099)
Institut Pierre-Simon Laplace (IPSL)	IPSL-CM5A-LR<br>IPSL-CM5A-MR<br>IPSL-CM5B-LR	Y<br>-<br>-	Y<br>Y<br>Y	Y*<br>-<br>-
Japan Agency for Marine-Earth Science and Technology, Atmosphere and Ocean Research Institute (The University of Tokyo), and National Institute for Environmental Studies	MIROC-ESM<br>MIROC-ESM-CHEM	Y<br>Y	Y<br>Y	Y*<br>Y*
Atmosphere and Ocean Research Institute (The University of Tokyo), National Institute for Environmental Studies, and Japan Agency for Marine-Earth Science and Technology	MIROC4h<br>MIROC5	Y<br>-	-<br>Y	-<br>-
Max Planck Institute for Meteorology (MPI)	MPI-ESM-LR<br>MPI-ESM-MR<br>MPI-ESM-P	Y<br>-<br>-	Y<br>Y<br>-	Y<br>-<br>-
Meteorological Research Institute (MRI)	MRI-CGCM3	Y	Y	Y*
Norwegian Climate Centre	NorESM1-M<br>NorESM1-ME	-<br>-	Y<br>Y	-<br>-

* These models also have 550 nm aerosol optical depth (AOD) data for historical and RCP8.5 periods

Table S2: Individual forcings runs from CMIP5 and CMIP3 (*in italics*) coupled models.

		Experiments							
	**Model**	**All**	**AA**	**NoAA**	**GHG**	**Nat**	**AsAA**	**RCP4.5**	**RCP8.5**
**CMIP5**	CanESM2	5	5	-	5	5	-	1	1
	CSIRO-Mk3.6	10	5	5	5	5	5	1	1
	GFDL CM3	5	3	-	2	3	-	1	1
	GISS-E2-R	6	5	-	5	5	-	1	1
	IPSL-CM5A-LR	3	1	4	6	3	-	1	1
**CMIP3**	*GFDL-CM2.1*	*5*	*3*	-	*3*	*3*	-	-	-
	PCM1	*3*	*4*	-	*4*	*4*	-	-	-
	**Total no. models**	**7**	**7**	**2**	**7**	**7**	**1**	**5**	**5**
	**Total no. runs**	**37**	**26**	**9**	**30**	**28**	**5**	**5**	**5**

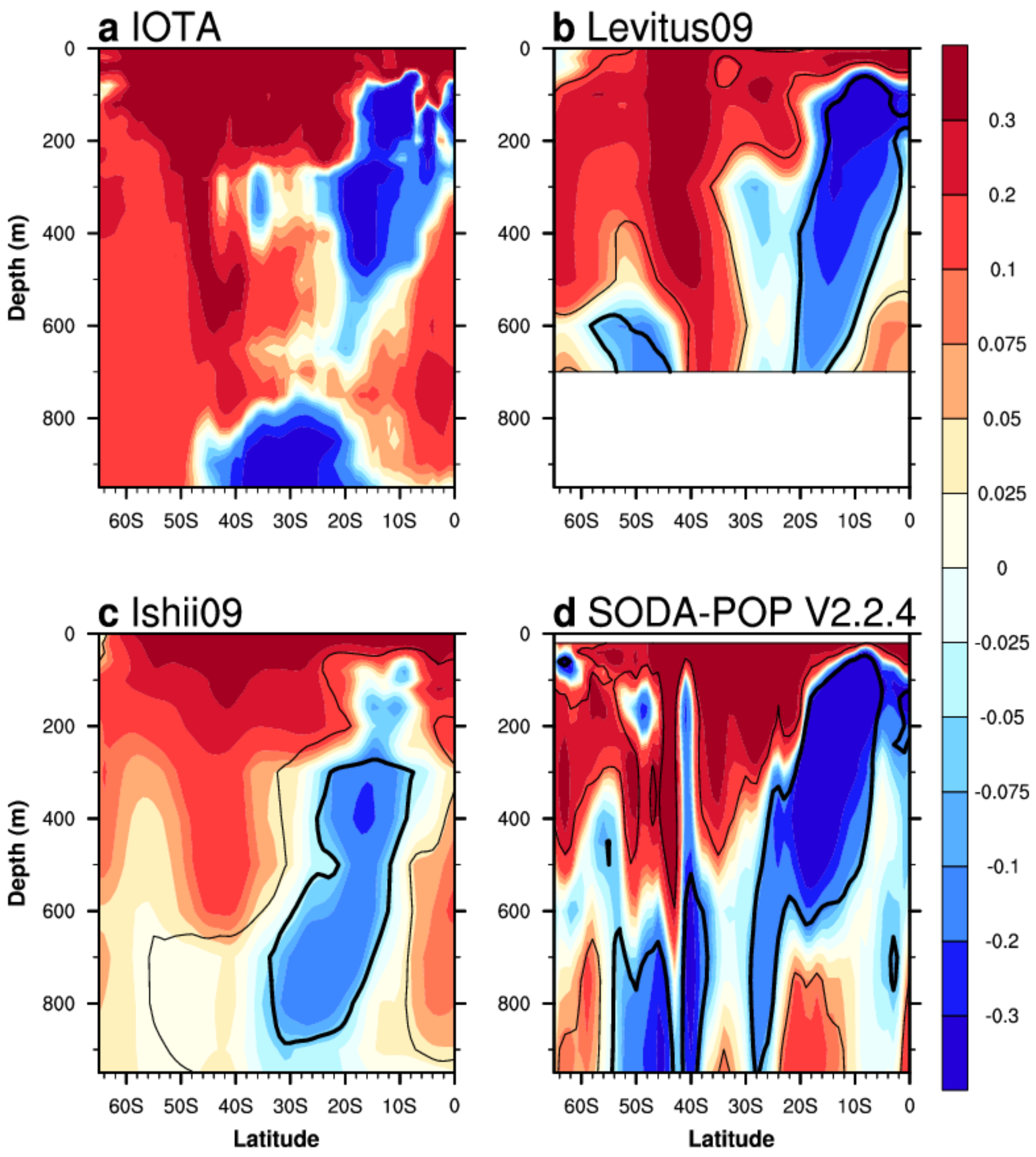

Figure S1| Observed zonal-mean linear subsurface temperature trends for the Indian Ocean. Zonally-averaged linear trends over 1960-1999 of subsurface temperature in the southern tropical and subtropical Indian Ocean (40°E-110°E), based on observational estimates from: (**a**) Indian Ocean Thermal Archive (IOTA), (**b**) Levitus temperature from the World Ocean Database 2009 (Levitus09), (**c**) Ishii temperature assimilation (Ishii09), and (**d**) Simple Ocean Data Assimilation- Parallel Ocean Program version 2.2.4 (SODA-POP_V2.2.4). Trend units are °C 40-years^{-1}. The Levitus09 observations are confined to the upper 700 m only. Significant trends at the 95% confidence level, based on a *t*-test, are shown within the contours for (b)-(d). For IOTA, no information on statistical significance was available at the time of writing.

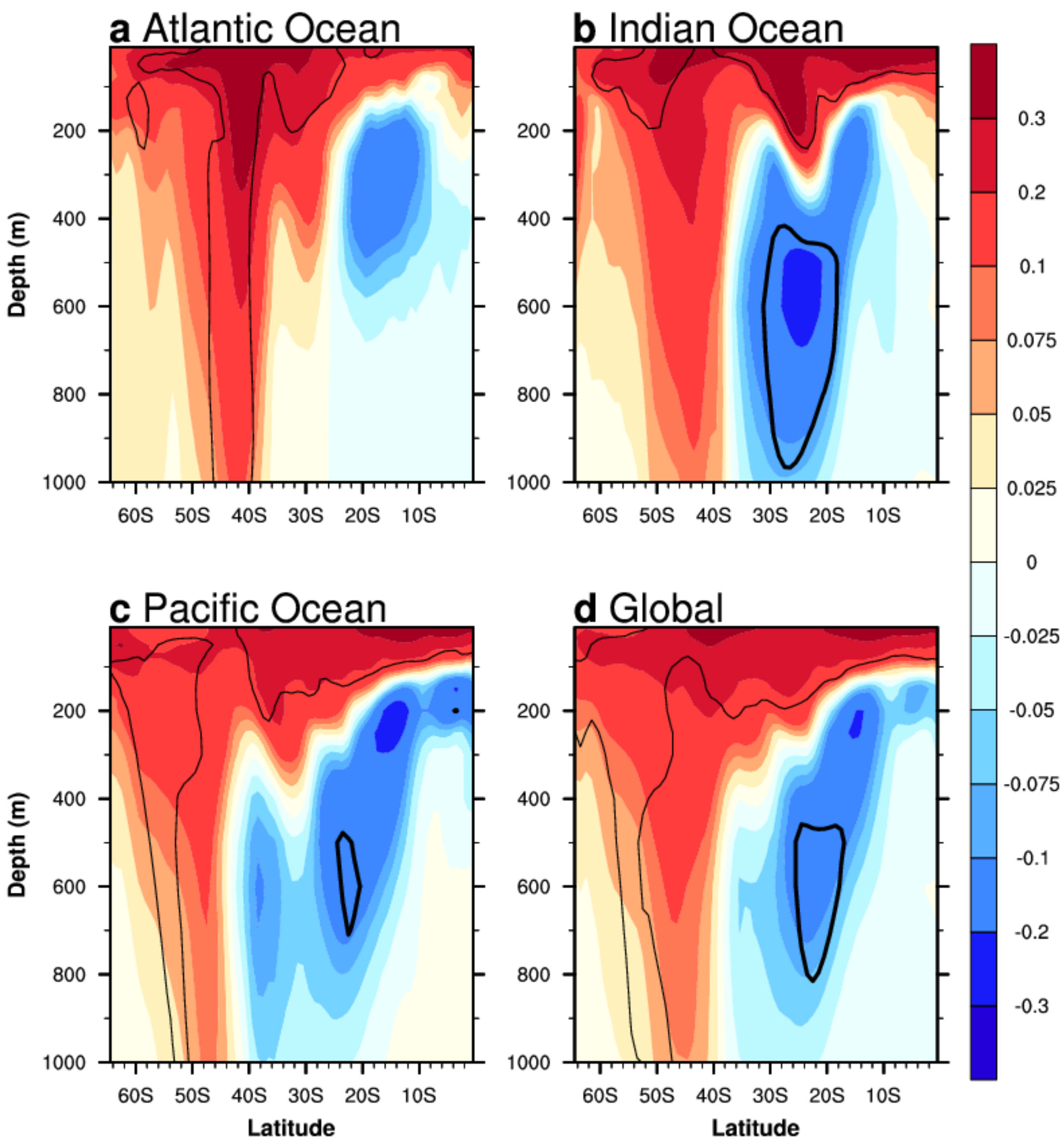

Figure S2| Simulated (de-drifted) zonal-mean linear subsurface temperature trends for each ocean basin. Zonally-averaged linear trends over 1960-1999 of subsurface temperature in the SH tropical and subtropical ocean basins based on a 15 CMIP5 multi-model ensemble that has been de-drifted: (**a**) Atlantic Ocean, (**b**) Indian Ocean, (**c**) Pacific Ocean, and (**d**) global ocean. Trend units are °C 40-years^{-1}. Significant trends, greater than one standard deviation of the inter-model spread, are shown within the contours.

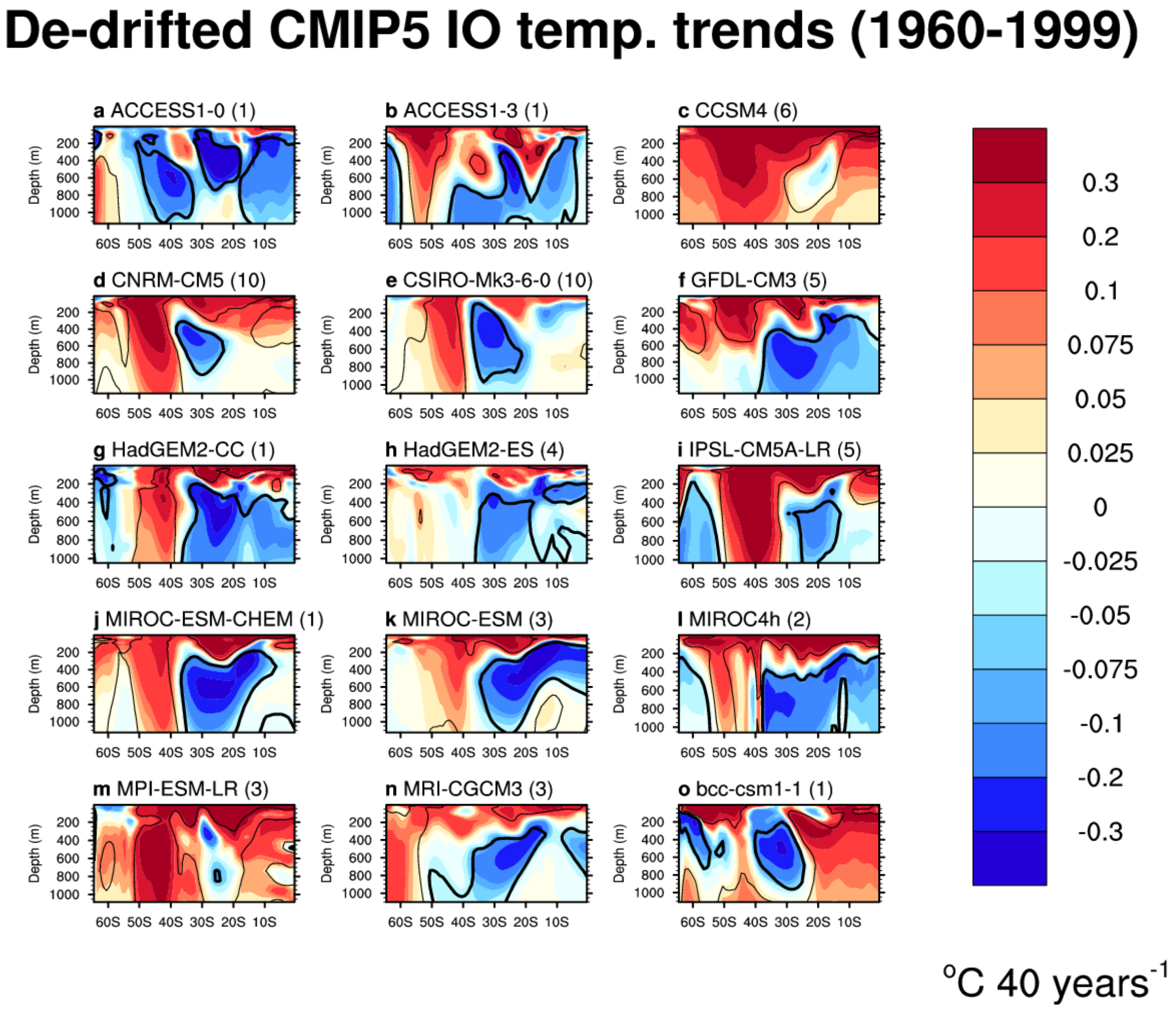

Figure S3| Simulated (de-drifted) zonal-mean linear subsurface temperature trends for the southern IO for each model ensemble. Zonally-averaged linear trends over 1960-1999 of subsurface temperature in the SH tropical and subtropical IO for each of the 15 CMIP5 models that have been de-drifted. Trend units are °C 40-years^{-1}. Significant trends at the 95% confidence level, based on a *t*-test, are shown within the contours. The number of model runs that make up each model ensemble is listed in the brackets in each panel title.

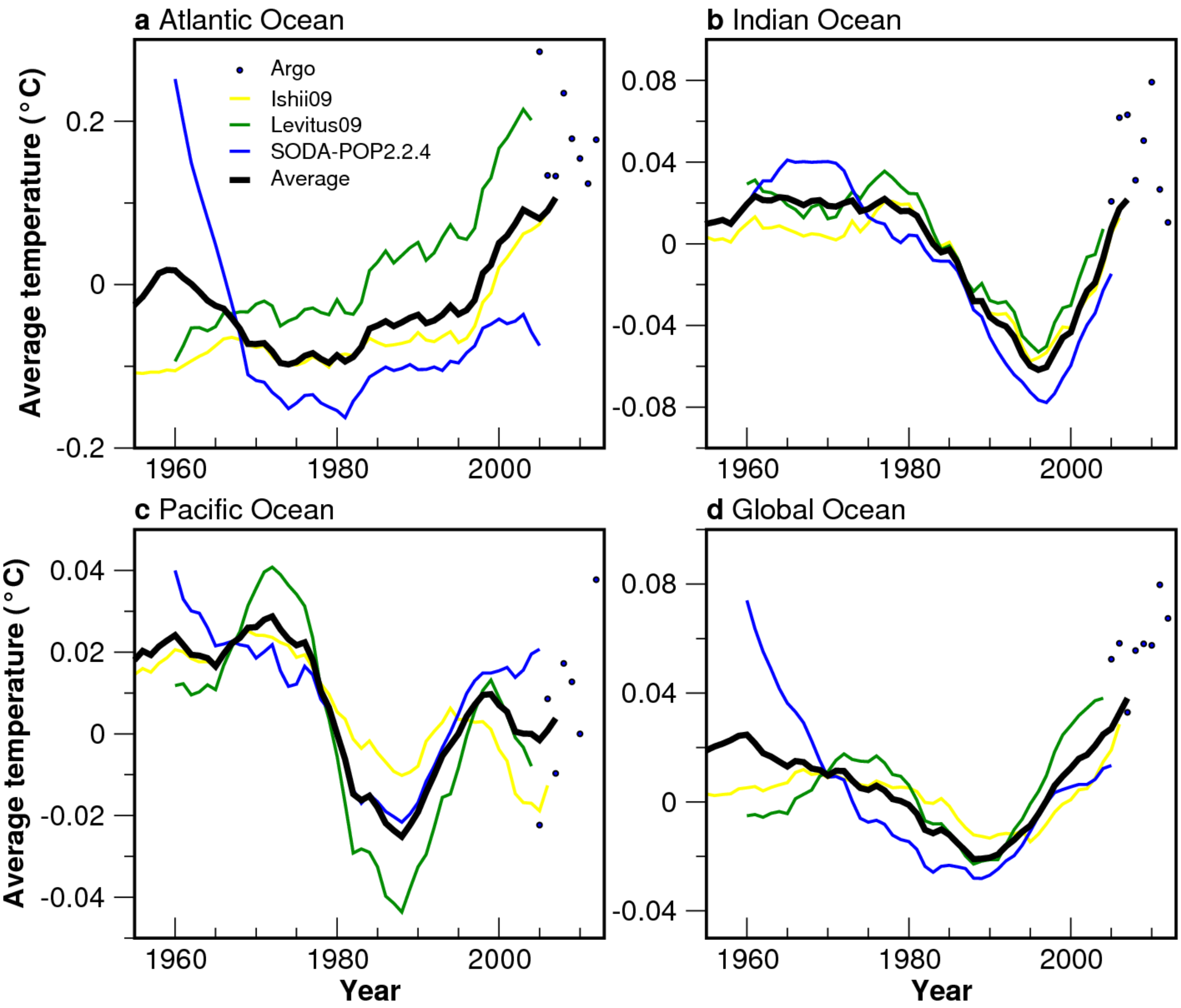

Figure S4| Average observed sub-thermocline temperature from the SH tropical and subtropical oceans. Mean sub-thermocline temperature from observational estimates for: (**a**) Atlantic Ocean (70°W-20°E, 20°S-40°S), (**b**) Indian Ocean (40°E-110°E, 10°S-30°S), (**c**) Pacific Ocean (120°E-70°W, 10°S-40°S), and (**d**) global ocean (10°S-40°S). The depth over which the average is taken is 300-900 m (except for Levitus09 which is 300-700 m). The observational estimate average (black line) is based on an average of the four products: Levitus09, Ishii09m, SODA-POP V2.2.4, and Argo profiles (2005-2012). Note the different temperature axis scale used in (**a**)-(**d**). All time series have been low-pass filtered using an 11-year running mean, except for the Argo measurements which show the interannual variability.

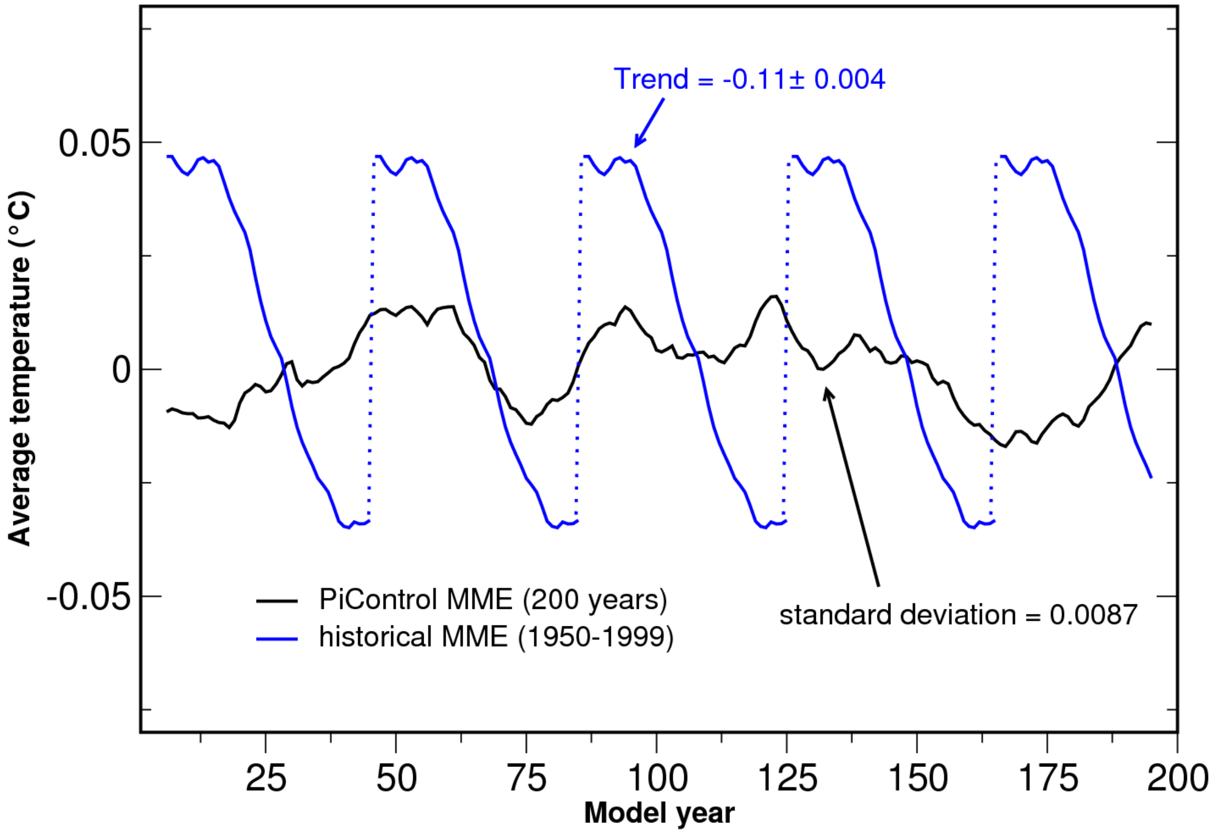

Figure S5| Significance of the simulated CMIP5 de-drifted MME time series. Comparison between the time series of sub-thermocline IO temperature from a 15-CMIP5 de-drifted MME (blue line) and a 15-CMIP5 MME pre-industrial control period (200-years; black line). The historical MME time series is shown five times, as a way to illustrate the historical change relative to the pre-industrial MME. All time series have been low-pass filtered using an 11-year running mean.

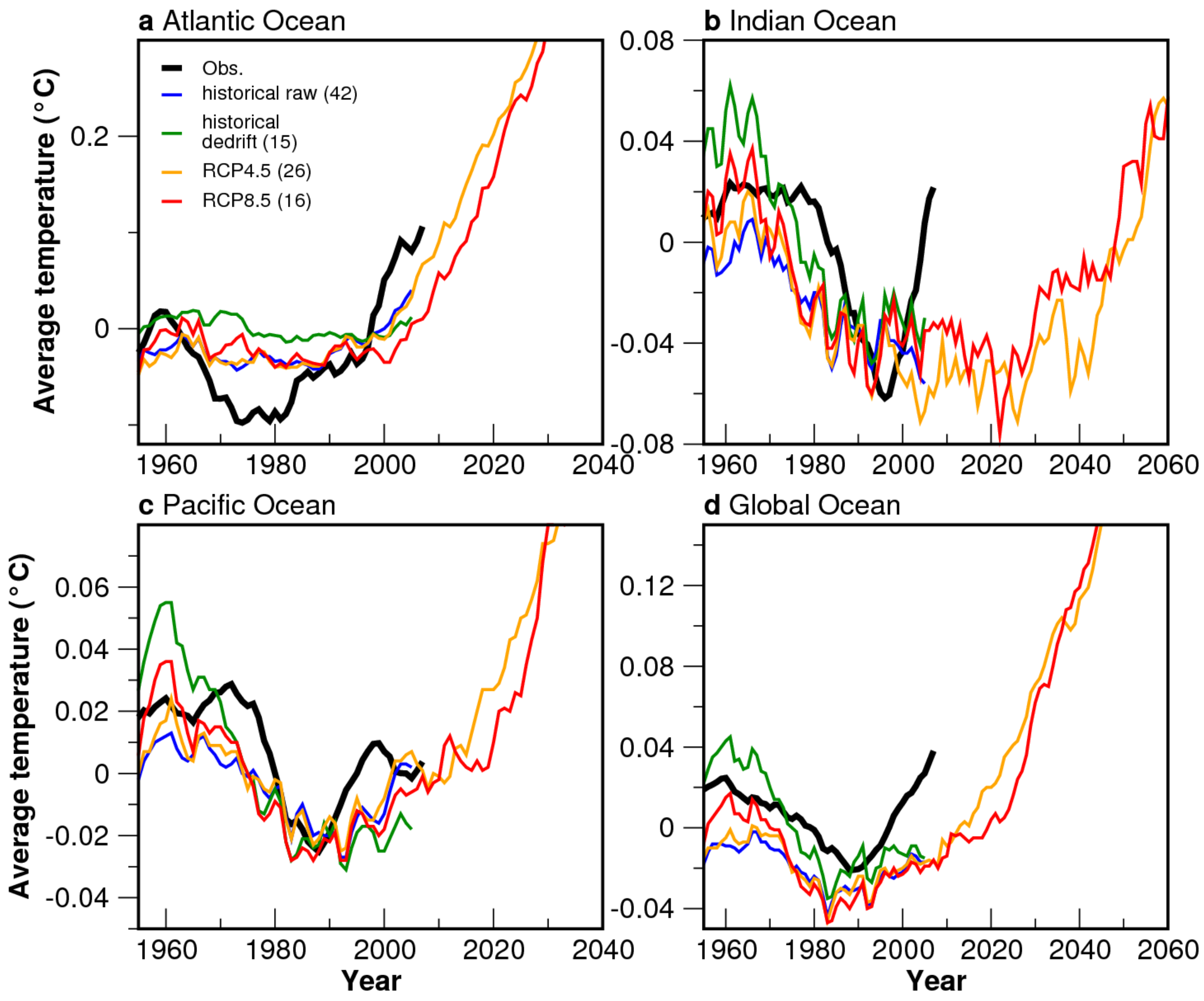

Figure S6| Average simulated sub-thermocline temperature from the SH tropical and subtropical oceans. Mean subsurface temperature from CMIP5 multi-model ensembles estimates for: (**a**) Atlantic Ocean (70°W-20°E, 10°S-30°S), (**b**) Indian Ocean (40°E-110°E, 15°S-35°S), (**c**) Pacific Ocean (120°E-70°W, 15°S-40°S), and (**d**) global ocean (15°S-35°S). The depth over which the average is taken is 300-900 m. The simulated estimates include a 42 CMIP5 historical MME (blue line), a 15 CMIP5 historical de-drifted MME (green line), a 26 CMIP5 RCP4.5 MME (orange line), and a 16 CMIP5 RCP8.5 MME (red line). All time series are shown as annual values, except for the observation averages (black line) which have been low-pass filtered using an 11-year running mean.

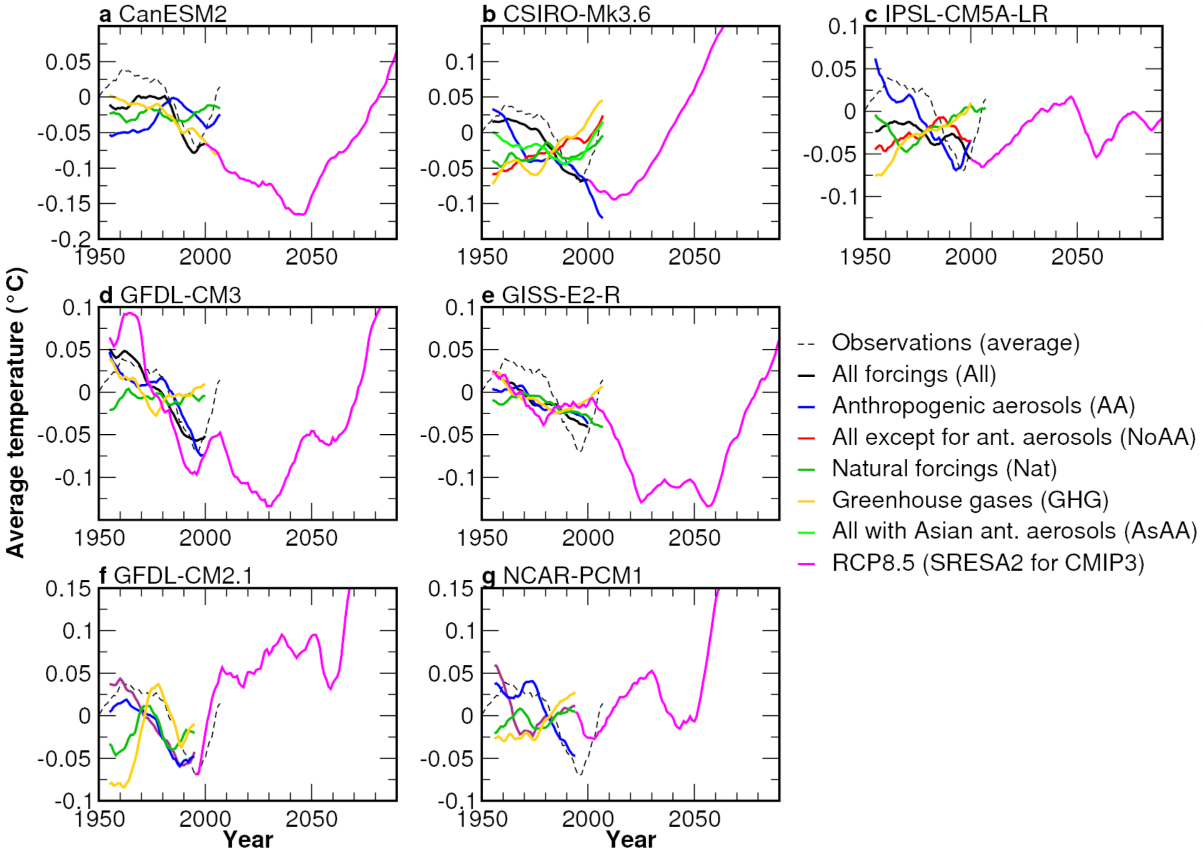

Figure S7| Average simulated sub-thermocline temperature from individual CMIP5 and CMIP3 models for the southern IO. Mean sub-thermocline IO temperature from model individual forcing runs (based on Table 2) for five CMIP5 models: (**a**) CanESM2, (**b**) CSIRO-Mk3.6, (**c**) IPSL-CM5A-LR, (**d**) GFDL-CM3, (**e**) GISS-E2-R; and two CMIP3 models: (**f**) GFDL-CM2.1, and (**g**) NCAR-PCM1. The region of interest is 40°E-110°E, 15°S-35°S, 300-900 m. Please note, the vertical axis is different between the individual models. All time series have been low-pass filtered using an 11-year running mean.

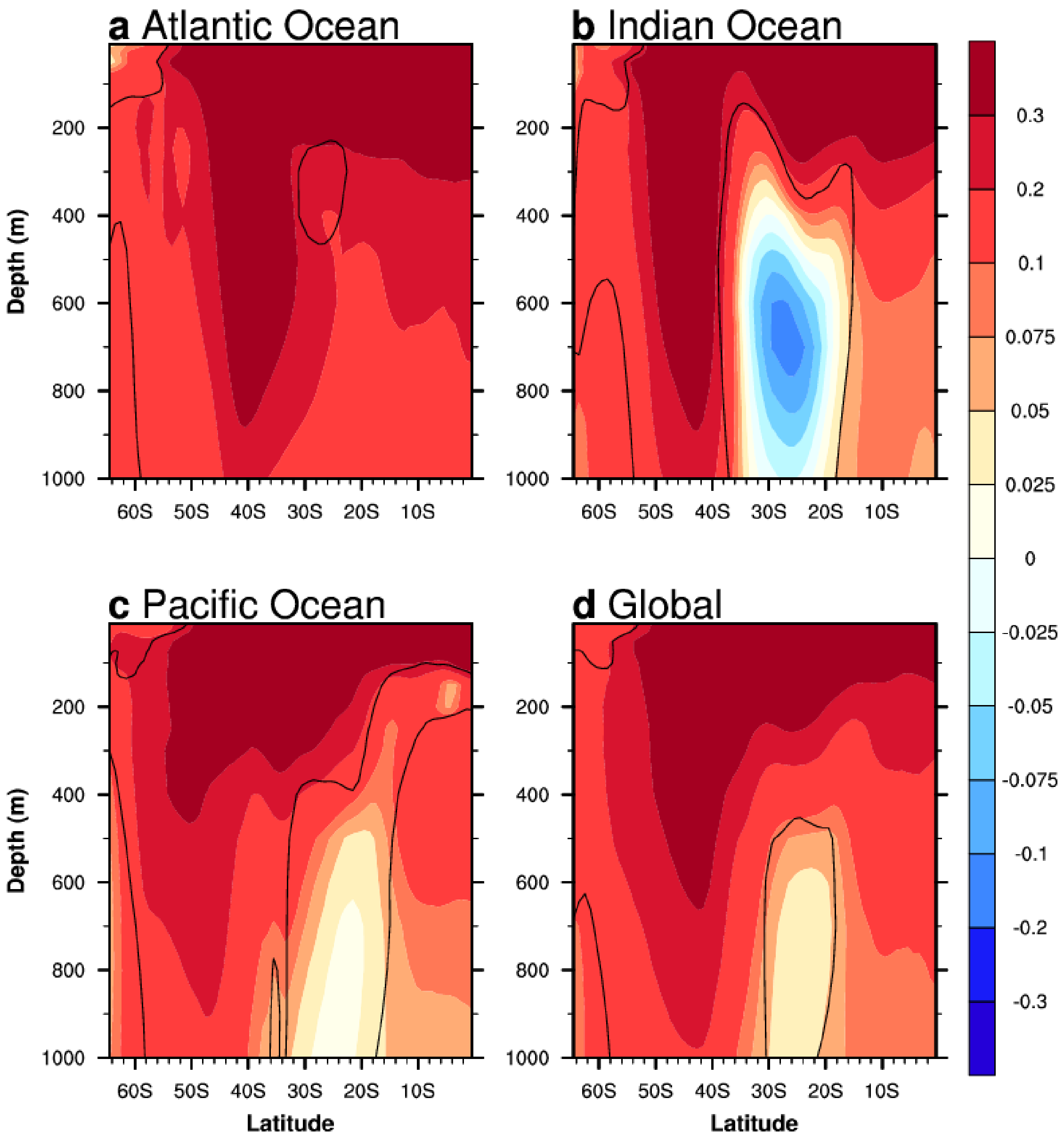

Figure S8| Simulated zonal-mean linear subsurface temperature trends for each ocean basin induced by greenhouse gases (GHG) only. Zonally-averaged linear trends over 1960-1999 of subsurface temperature in the SH tropical and subtropical ocean basins based on a MME of five CMIP5 and two CMIP3 models forced only by GHGs, for: (**a**) Atlantic Ocean, (**b**) Indian Ocean, (**c**) Pacific Ocean, and (**d**) global ocean. Trend units are °C 40-years^{-1}. Significant trends, greater than one standard deviation of the inter-model spread, are shown within the contours.

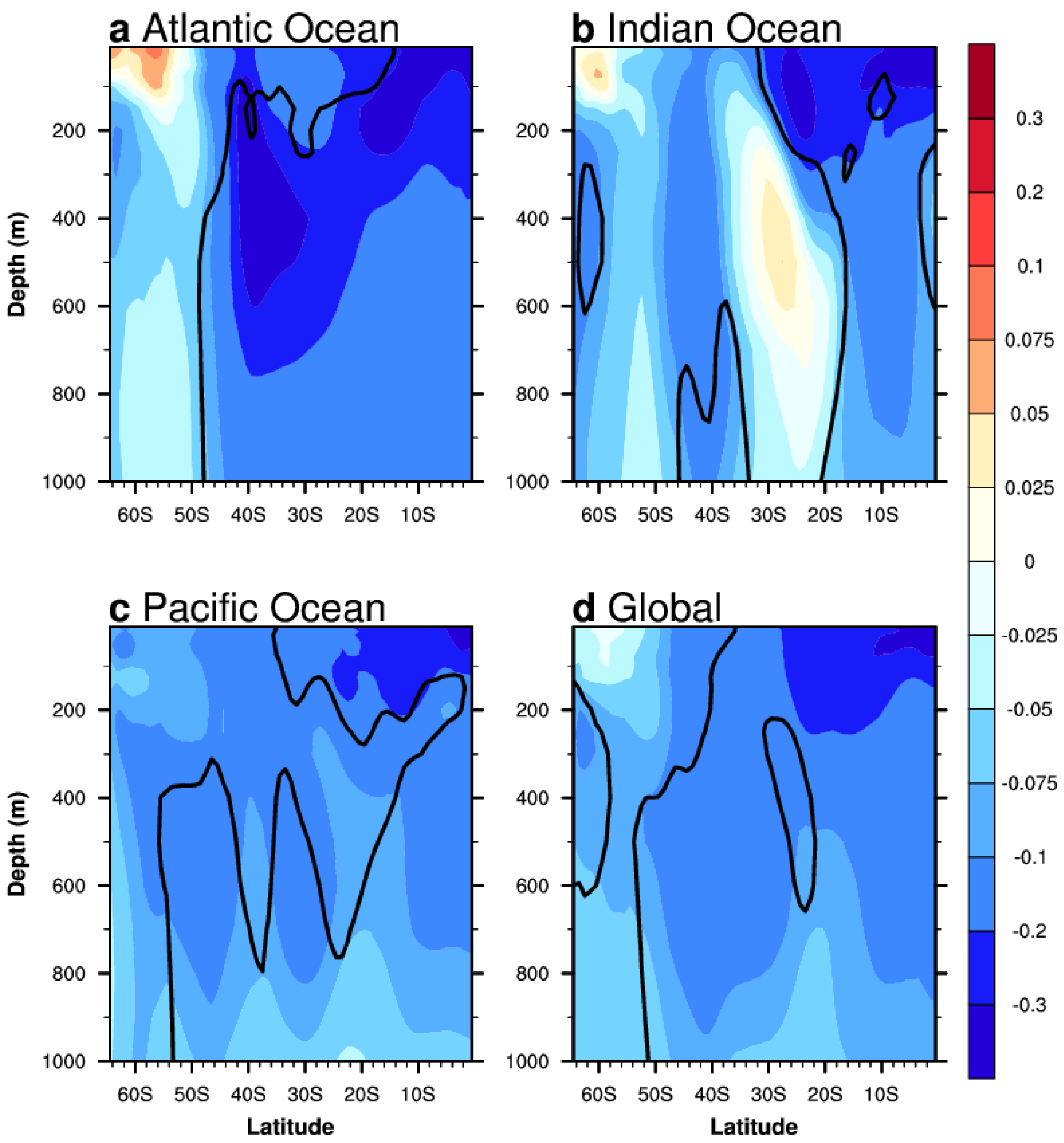

Figure S9| Same as Figure S8, but for models forced by anthropogenic aerosols (AA) only.

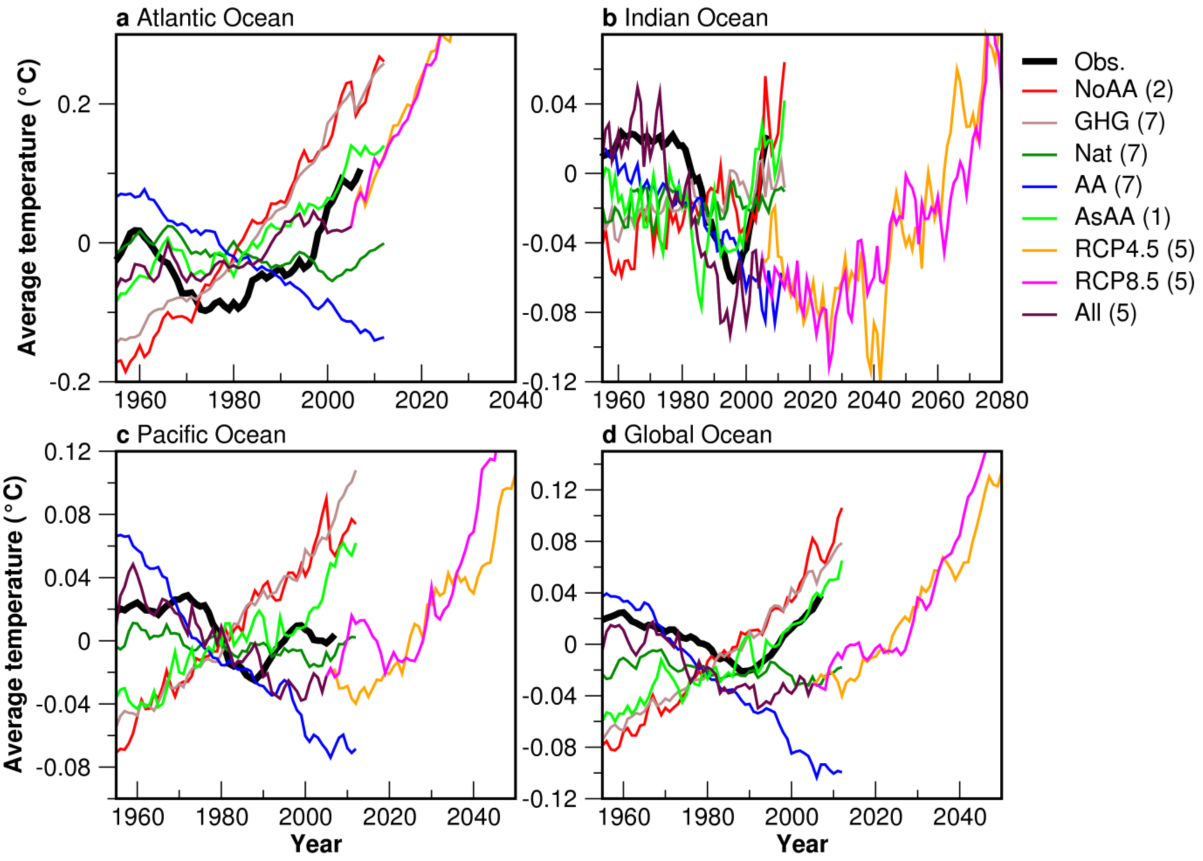

Figure S10| Average simulated sub-thermocline temperature from individual forcing runs. Mean sub-thermocline temperature from model individual forcing runs (based on Table 2) for: (**a**) Atlantic Ocean (70°W-20°E, 10°S-30°S), (**b**) Indian Ocean (40°E-110°E, 15°S-35°S), (**c**) Pacific Ocean (120°E-70°W, 15°S-40°S), and (**d**) global ocean (15°S-35°S). The depth over which the average is taken is 300-900 m. The simulated annual estimates include a three-model NoAA ensemble (red line), an eight-model GHG ensemble (grey line), an eight-model Nat ensemble (dark green line), a seven-model AA ensemble (blue line), a two-model AsAA ensemble (light green line), a five-model All ensemble (maroon line), and five-model RCP4.5 (orange line) and RCP8.5 ensembles (pink line). The observational average (shown as an 11-year running mean) is also shown (black line). All time series are shown as annual values, except for the observation average which has been low-pass filtered using an 11-year running mean.

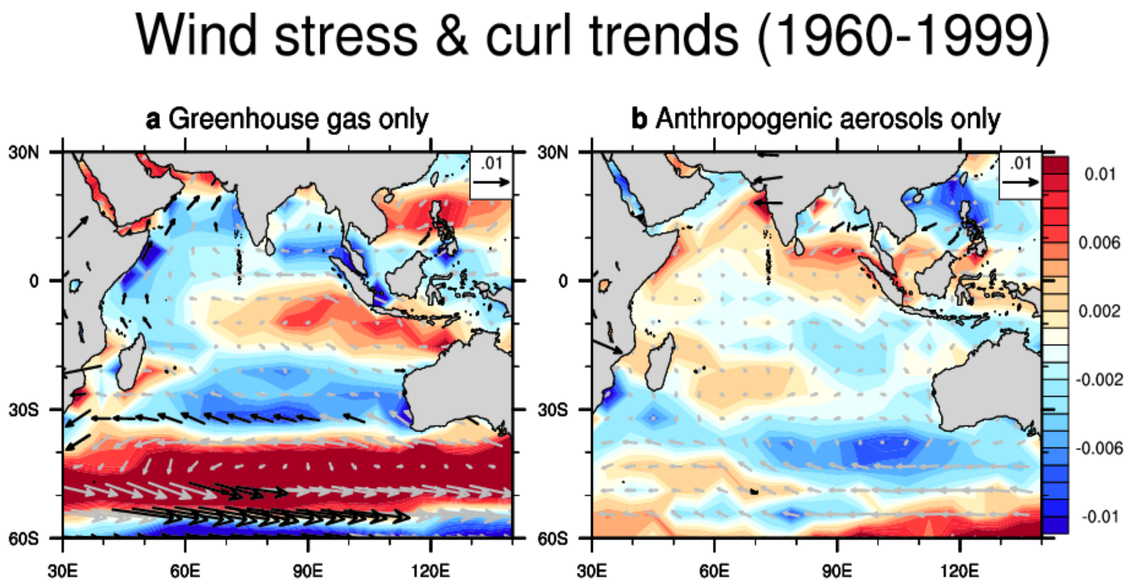

Figure S11| Linear trends over 1960-1999 of wind stress curl (contour) and wind stress (vectors) in the SH tropical and subtropical Indian Ocean based on a five CMIP5 multi-model ensemble forced only with: (**a**) well-mixed greenhouse gases and (**b**) anthropogenic aerosols only. Curl trend units are N m^{-3} 40-$years^{-1}$, scaled by 10^6, while the wind stress reference vector is 0.01 N m^{-2} 40-$years^{-1}$. Wind vectors that are black are those which are greater than the one-standard deviation of the intermodel spread.

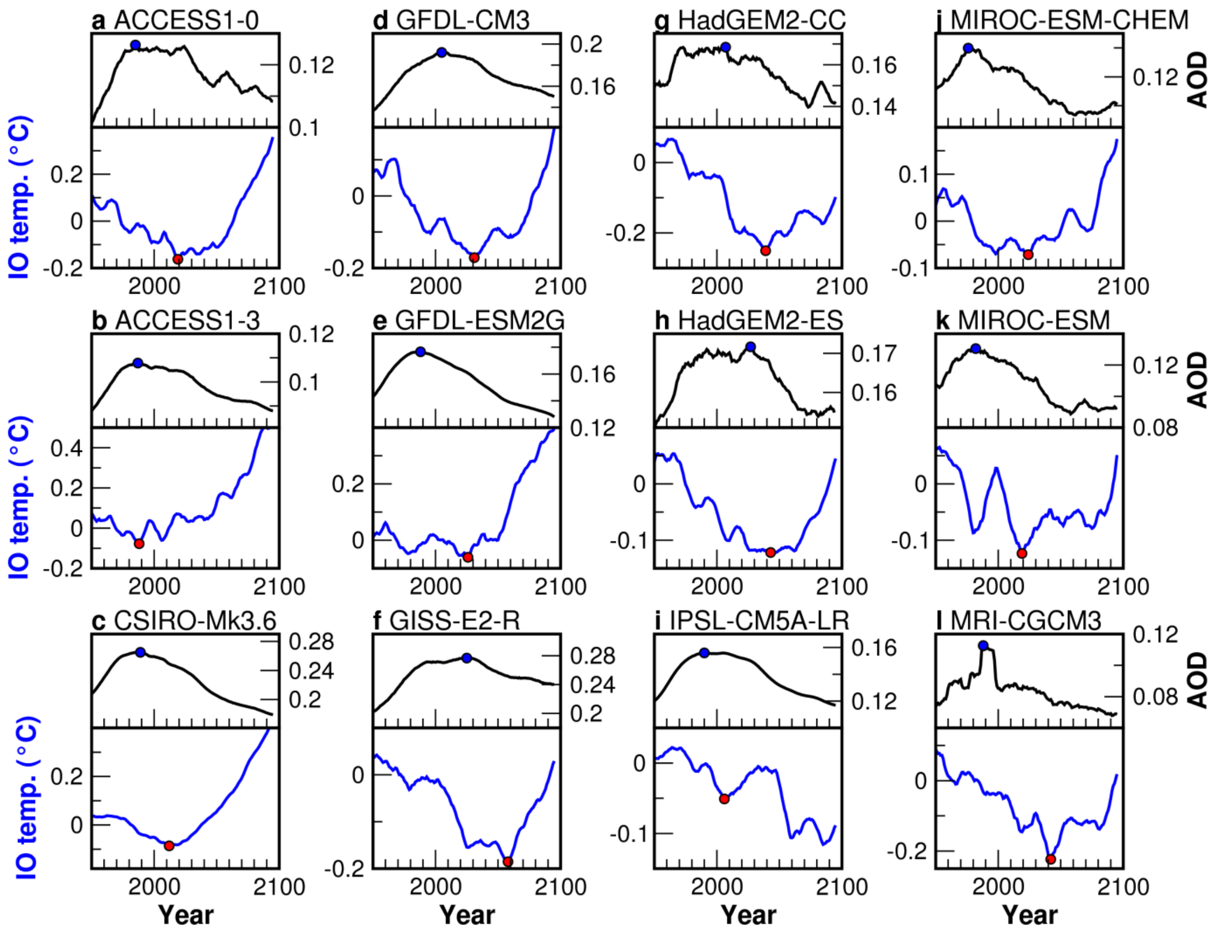

Figure S12| Simulated southern Indian Ocean sub-thermocline temperature versus aerosol optical depth. Average temperature of the southern sub-thermocline IO (bottom plots; blue line) versus aerosol optical depth (AOD) at 550 nm averaged over the NH (top plots; black line) from 1950-2100 (based on historical and RCP8.5 experiments), as simulated by: (**a**) ACCESS-1.0, (**b**) ACCESS-1.3, (**c**) CSIRO-Mk3.6, (**d**) GFDL-CM3, (**e**) GFDL-ESM2G, (**f**) GISS-E2-R, (**g**) HadGEM-CC, (**h**) HadGEM-ES, (**i**) IPSL-CM5A-LR, (**j**) MIROC-ESM-CHEM, (**k**) MIROC-ESM, and (**l**) MRI-CGCM3. The blue (red) dots indicate the peak (trough) in NH AOD (IO sub-thermocline temperature). CSIRO-Mk3.6, HadGEM-ES, IPSL-CM5A-LR are ensembles made up 10, four, and four runs, respectively, while the remaining models consist of one run only. All time series have been low-pass filtered using an 11-year running mean. It should be noted that the IPSL-CM5A-LR model does not show an absolute IO minimum, so it is taken as the minimum prior to the multi-decade warming post-2010.

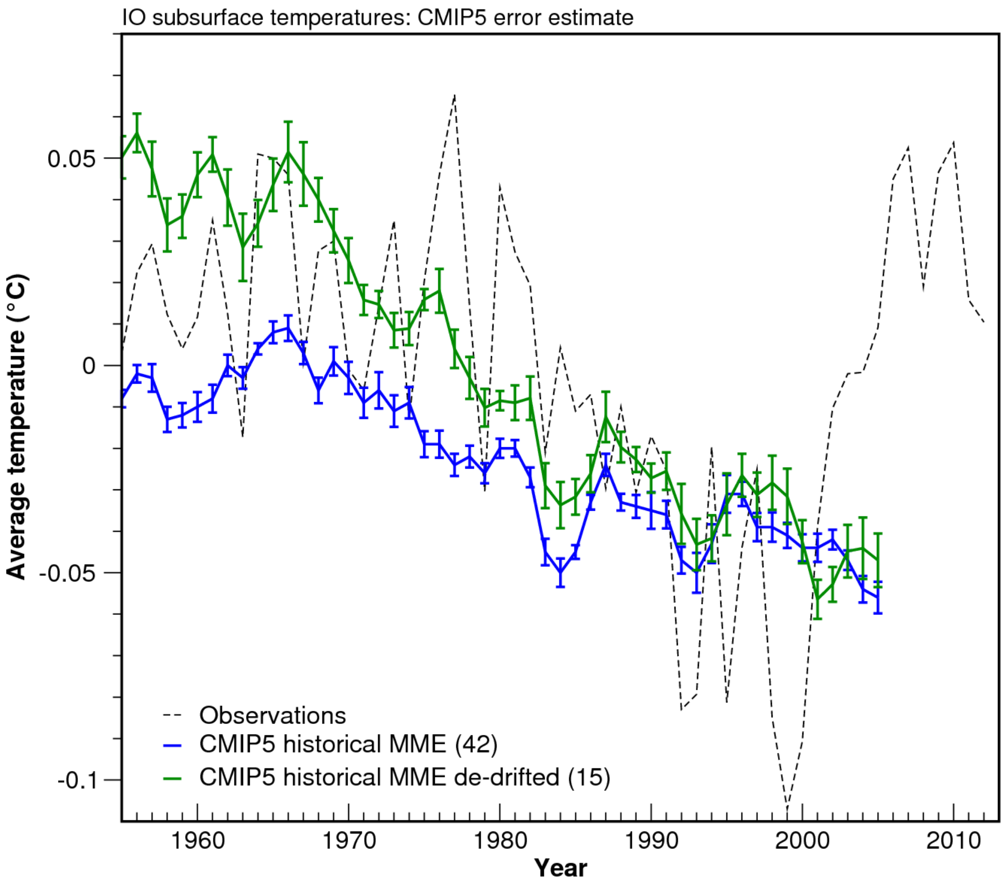

Figure S13| Average simulated sub-thermocline temperature from the southern IO with error bars. Mean sub-thermocline temperature for the IO (40°E-110°E, 15°S-35°S, 300-900 m). The simulated annual estimates include a 42 CMIP5 historical MME (blue line) and a 15 CMIP5 historical de-drifted MME (green line). The annual observed average is also shown (black dotted line). The error-estimates are the 95% confidence interval, and are based on the statistics of 10 MMEs using one run selected randomly from each model, repeated 10 times to create the desired 10 member ensemble. For models with only one run, this run is used each time in the 10 model ensemble average.
